# Regulation of the bone marrow microenvironment by G-CSF: Effects of G-CSF on acute lymphoblastic leukaemia

**DOI:** 10.1371/journal.pone.0188042

**Published:** 2017-11-16

**Authors:** Jordan Basnett, Vicki Xie, Adam Cisterne, Ken Bradstock, Linda Bendall

**Affiliations:** 1 Centre for Cancer Research, The Westmead Institute for Medical Research, University of Sydney, Sydney, New South Wales, Australia; 2 Department of Haematology, Westmead Hospital, Sydney, New South Wales, Australia; European Institute of Oncology, ITALY

## Abstract

It has been suggested that disruption of the lymphoid niche by G-CSF may be of therapeutic benefit to patients with acute lymphoblastic leukaemia. We used a xenograft model to determine the effect of G-CSF on ALL progression in a minimal residual disease setting. Consistent with the effects on normal murine B cell progenitors, G-CSF slowed disease in the majority of ALL xenografts tested, suggesting that G-CSF may provide benefits beyond neutrophil recovery for ALL patients. However, two of eight xenografts demonstrated accelerated disease progression. G-CSF could be detrimental for these patients due to expansion of the malignant clone.

## Introduction

Interfering with micro-environmental support to augment the efficacy of anti-leukaemic therapies has been of considerable interest over the last decade. Most recently G-CSF has been shown to disrupt lymphopoiesis in mice resulting in a dramatic loss of normal B cell progenitors from the bone marrow, which is not accounted for by mobilization of these cells into the periphery [1]. Acute lymphoblastic leukaemia (ALL) cells, like their normal counterparts, are highly dependent on micro-environmental support for optimal growth [2, 3]. This raised the possibility that the administration of G-CSF to ALL patients may be of therapeutic benefit.

A recent analysis of long-term follow-up data from 5 European clinical trials examining the impact of prophylactic G-CSF revealed survival advantages, particularly extensions in disease free survival (p = 0.01) and remission duration (p = 0.007), in the G-CSF group [4]. The authors proposed that this was the result of improved chemotherapy dose delivery, although this was not directly measured in this study and it remains possible that disturbances to the microenvironment contributed to the improved survival in G-CSF treated patients. A more recent study by Uy et al investigated the micro-environmental disruptive effects of G-CSF in patients with relapsed/refractory ALL. This study showed that the microenvironment was already significantly disturbed in patients with high disease burden although bone marrow supportive factors such as CXCL12 could be further suppressed by G-CSF administration and this was associated with increased apoptosis in ALL cells in the bone marrow [5].

## Materials and methods

### Xenograft model

All xenografts were previously established [6–9] from diagnostic bone marrow aspirates collected prior to the commencement of treatment. Cells recovered from the spleens of xenografted mice were used in these studies. All procedures were approved by the Westmead Hospital Animal Ethics Committee. Six to 8 week old female NOD/SCID (bred in house) or NSG (Australian BioResources Pty Ltd, Australia) mice were acclimatized and subsequently housed in IVC racks with sterile water food and bedding for 1 week prior to receiving 3-5x10^6^ ALL cells by tail vein injection. When the bone marrow contained approximately 1% leukemia, mice were treated twice daily with G-CSF (125 μg/kg) by subcutaneous injection (Amgen, Thousand Oaks, CA, USA) for 10 days then culled by CO_2_ asphyxiation. No symptoms of disease or treatment were evident in this time period. Blood was collected by cardiac puncture and bone marrow flushed from 1 femur with 1 mL of Roswell Park Memorial Institute (RPMI) medium containing 10% FCS (RF-10). A single cell suspension was prepared from complete spleens using a 40 μm cell strainer with 10 mL of RF-10. ALL cells in each tissues was enumerated by flow cytometry using mouse anti-human CD19-PE (cat:302208, clone HIB19, Biolegend, San Diego, CA, USA) and rat anti-mouse CD45-FITC (cat:553080, clone 30-F11, BD Biosciences). Where necessary red blood cells were removed by incubation with 1 mL of red cell lysis buffer (0.15 M NH_4_Cl, 0.01 M KHCO_3_, and 0.01 mM EDTA) at room temperature for 10 min.

### Flow cytometry

Cells were labelled with indicated antibodies for 10 min at room temperature and washed with phosphate buffered saline (PBS), containing 0.1% sodium azide and 0.1% bovine serum albumin (BSA), by centrifugation at 365 x *g* for 5 min. The supernatant was discarded and the pellets resuspended in 90 μL PBS and 10 μL 7-AAD (cat:559925, BD Biosciences). Samples were analysed on a BD FACScanto flow cytometer.

### G-CSF receptor expression

Cells were labelled as described above using the mouse anti-human CD114 PE (cat:554538, BD Biosciences, North Ryde, NSW, Australia). G-CSF receptor expression was evaluated on viable ALL cells by flow cytometry.

### Bone marrow stroma

Bone marrow cells were flushed from femurs and/or tibias of mice and cultured in Minimum Essential Medium Eagle α modification (**α**-MEM) (Sigma, Castle Hill, NSW, Australia) supplemented with 12.5% FCS (Sigma), 12.5% horse serum (Sigma), 4 mM L-glutamine (Invitrogen, Mulgrave, VIC, Australia), 50 U/ml penicillin (Invitrogen), 50 U/ml streptomycin (Invitrogen) and 10^−6^ M hydrocortisone (Sigma) until confluent layers were obtained. The immortalised human bone marrow stromal cell line DC.hTERT, kindly provided by Dario Campana (St Judes, Memphis, USA) was cultured in RF-10. Stromal cultures were passaged as required using trypsin/EDTA (Invitrogen) as previously described [10].

### ALL cell culture

ALL cells (1x10^6^) were plated in triplicate into wells containing confluent stromal layers where indicated, and G-CSF added at 10 or 50 ng/mL. Cells were harvested using trypsin/EDTA and viable ALL cell number analysed by flow cytometry using mouse anti-human CD19-APC (cat:557835, clone SJ25C1, BD Biosciences) or CD45-FITC (cat:347463, clone 2D1, BD Biosciences), to identify ALL cells and 7-AAD exclusion to determine viability.

## Results and discussion

**Fig 1:**
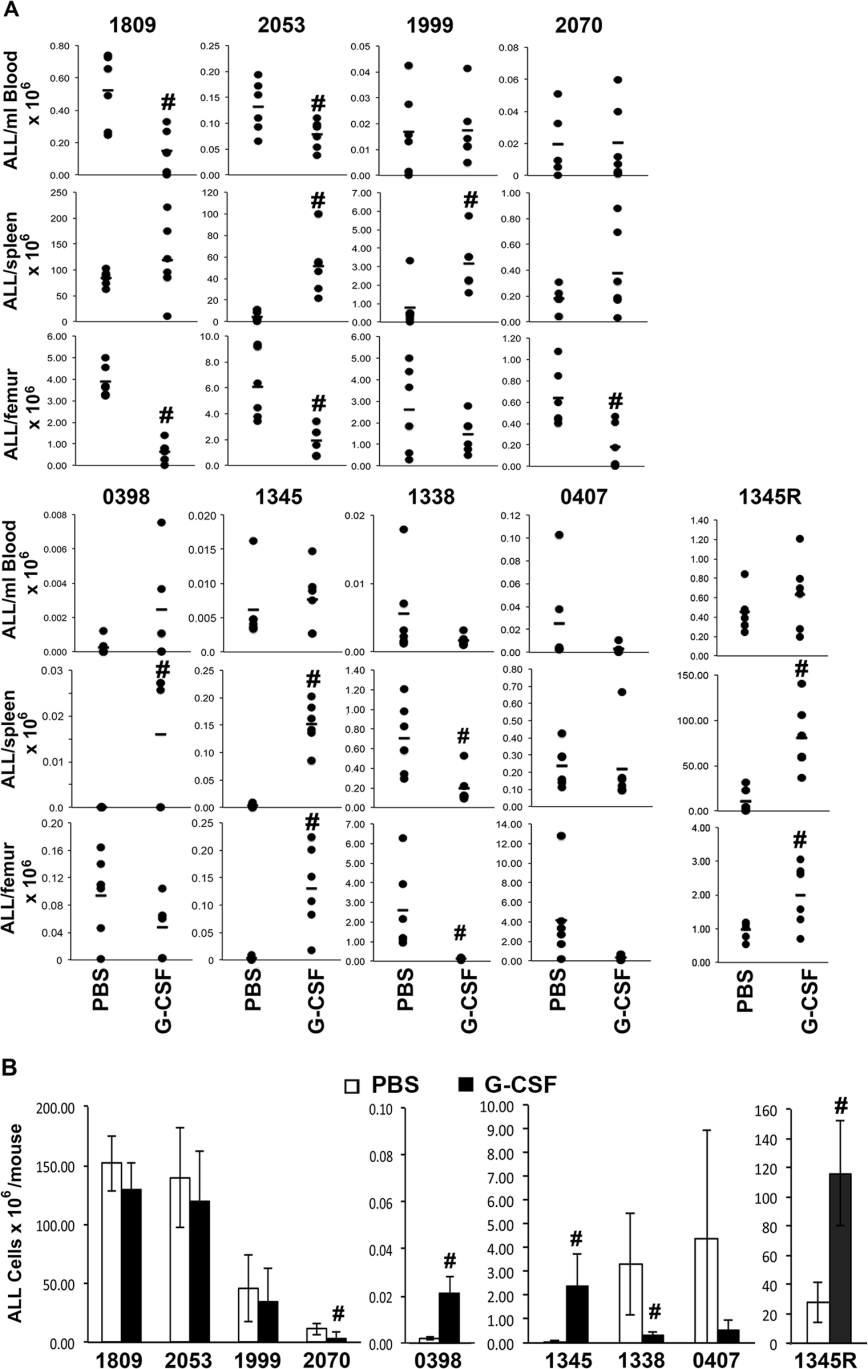
Effect of G-CSF on the development of human ALL in immunocompromised mice. Mice engrafted with the indicated ALL xenografts (1345R is a repeat of 1345 where initiation of treatment was delayed by 10 days) were treated with G-CSF (125 μg/kg twice daily) for 10 days and culled. **(A)** The number of ALL cells detected in the indicated tissue is indicated. Each dot represents an individual animal and the mean of the group is indicated by the bar. **(B)** The mean±SD of the total leukaemia detected in animals is shown. Open bars are control and black bars G-CSF treated. # p<0.05.

We used a NOD/SCID xenograft model of human ALL [6] to determine whether disturbances to the bone marrow microenvironment resulting from administration of G-CSF (filgrastim, Amgen, Thousand Oaks, CA, USA) could influence the progression of ALL *in vivo*. These studies were conducted with approval from the Westmead Hospital Animal Ethics Committee. A total of 8 patient xenografts were examined using groups of 6 NOD/SCID mice in each treatment group. Engrafted mice received G-CSF or PBS for 10 days commencing when disease in the bone marrow was predicted to be 1% based on preliminary studies (Fig A in S1 Text). All mice displayed elevated white cell counts following 10 days of G-CSF (mean of 12.0±5.0x10^9^/L up from 3.2±1.0x10^9^/L, n = 6). The effect of G-CSF on disease in the blood, bone marrow and spleen varied considerably between individual patient xenografts. The percentage of ALL cells in the blood was significantly reduced in 3 xenografts (1345, 1809 & 2053) and an additional 4 (1999, 2070, 0407 & 1338) demonstrated a trend towards decreased ALL contribution. However, this was significantly influenced by the increase in total WBC due to the expected myeloid expansion of murine cells in response to G-CSF. When the absolute number of ALL cells/μl was assessed only 2 xenografts (1809 & 2053) displayed significantly reduced numbers, while xenograft 0398 demonstrated increased numbers of circulating ALL cells; in 5 other xenografts number were unchanged (Fig 1A). Increased peripheral leukaemia cells could result from mobilization from the bone marrow, and in 4 (0398, 1345, 1999 & 2053) of the 8 xenografts an increased percentage and overall number of ALL cells in the spleens of animals suggested that this was occurring in some cases. This provides support for earlier anecdotal observation of ALL mobilization in response to G-CSF [11]. Bone marrow disease was significantly reduced in 4 of 8 xenografts (1809, 2070, 2053 & 1338) with a trend towards lower disease levels another 3 (0398, 1999 & 0407). Reduced disease could result from mobilization of ALL cells into the periphery, or reduced leukaemic cell proliferation or increased death secondary to marrow micro-environmental disruption, similar to that observed for normal B cell progenitors [1]. In contrast, xenograft 1345 demonstrated a significant increase in bone marrow disease.

Due to the variability of responses in individual tissues, we calculated the total disease in each animal based on 1 femur containing 5.8% of the bone marrow and the total blood volume being 80 ml/kilogram of mouse [12] and the contents of the spleen. Half of the xenografts (1809, 1999, 2053 & 0407) demonstrated a small but insignificant reduction in overall disease in response to G-CSF, while xenografts 2070 and 1338 showed a clear 68% (p<0.006) and 92% (p<0.007) decrease in ALL respectively (Fig 1B). In contrast, xenografts 0398 and 1345 displayed significantly increased disease of 10.5 (p = 0.02) and 34.6 (p = 0.0007) fold respectively. While the reduced disease seen in 6 of the xenografts is consistent with responses of normal B cell progenitors, the increase in the remaining 2 was unexpected and suggests that G-CSF may increase disease progression in a subset of patients. We noted that both xenografts where increased disease had been observed had lower levels of BM engraftment. To determine whether this was responsible for the difference in response we repeated the experiment using 1345 but delayed the initiation of treatment by 10 days to increase the level of engraftment. While engraftment levels were now higher, G-CSF treatment still increased disease burden (Fig 1A & 1B, 1345R).

**Fig 2:**
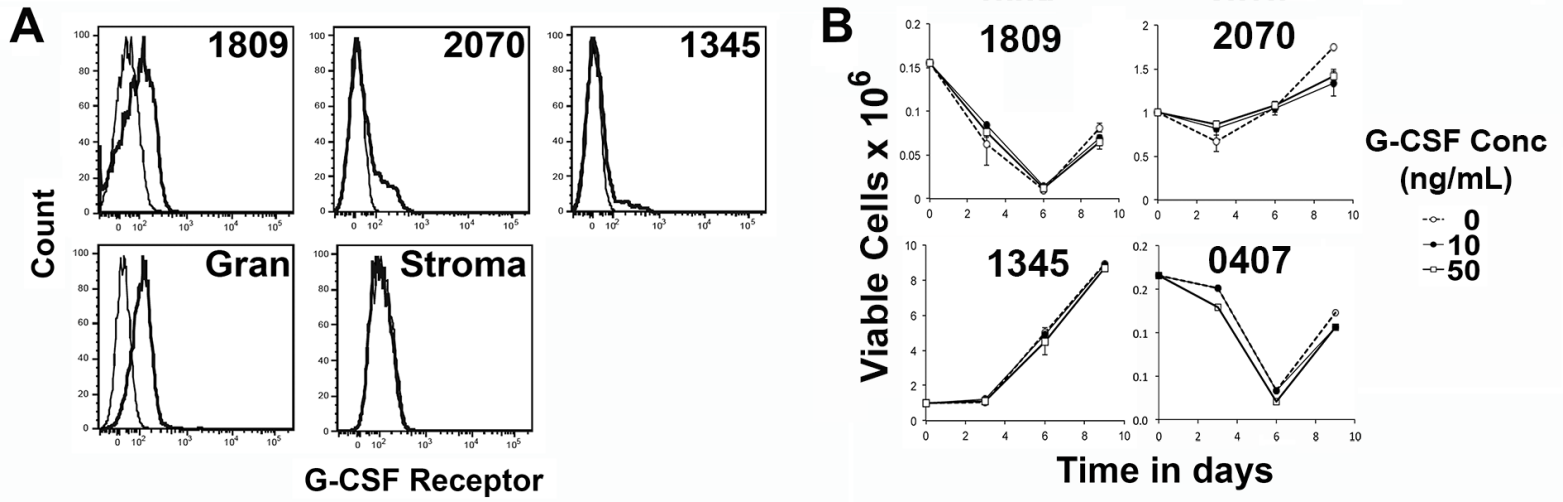
Effect of G-CSF on the in vitro growth of ALL xenograft cells. **(A)** Expression of the G-CSFR on ALL cells as determined by flow cytometry. The fine line is the isotype control and the heavy line the G-CSFR specific antibody. **(B)** The lack of response of ALL cells to G-CSF (added at 0, 10 or 50 ng/ml) in *in vitro* cultures in the presence of human (hTERT Stroma).

ALL cells have been variably reported to express the G-CSF receptor and respond to exogenous G-CSF [13, 14]. We examined the cell surface expression of the G-CSF receptor (G-CSFR) on ALL cells by flow cytometry to determine whether the differences in the *in vivo* responses could be explained by direct proliferative and survival responses of the ALL cells to G-CSF. Most samples displayed a small shoulder population suggesting that a subset of cells express low levels of the receptor (Fig 2A and data not shown). In contrast, sample 1809 displayed significant expression of G-CSFR, approaching that observed on normal neutrophils (Fig 1A). However, there was no association between G-CSFR expression and the *in vivo* response of the xenografts, with 1809 having high receptor expression but a slight reduction in disease, while 1345, which had a dramatic expansion *in vivo*, showed minimal G-CSFR expression.

The lack of correlation with direct responses to G-CSF was confirmed using *in vitro* cultures of ALL cells from 6 patients that had been successfully expanded on human stroma. These cells were cultured either alone or in the presence of human or murine stromal support, with or without the addition of exogenous G-CSF. Viable cell recovery over a 9-day culture period failed to detect any significant effect of exogenous G-CSF on ALL cell expansion in any of the samples tested regardless of the presence of supportive murine stroma (propagated from bone marrow mononuclear cells flushed mouse bone marrow [15]) or the human stromal cell line DC.hTERT (Fig 2B and data not shown).

While the inhibitory effects of G-CSF on ALL cell expansion *in vivo* were less than expected, they were clearly evident in one patient xenograft (2070) and trends were present in an five additional xenografts (1809, 2153, 1999, 1338 and 0407). These findings are consistent with and at least potentially provide a partial explanation for the increased disease free survival and duration of remission of patients receiving G-CSF [4]. However, the unexpected and much more dramatic increase in disease in the remaining two xenografts is unexplained and of concern. It suggests that, although most patients are likely to benefit from the addition of G-CSF, for a minority G-CSF administration may significantly worsen clinical outcome, and this effect could easily be missed in clinical studies where the beneficial effects in the majority of patients would mask detection. If these patients could be identified then it would be possible to tailor G-CSF use to minimise this potential problem. However, the two patient samples where disease was increased following G-CSF administration did not have cytogenetic or clinical features that distinguished them from the other patient samples (Table 1 and Table A in S1 Text), and we have been unable to determine clinical or biological features of these cases that distinguish them from others that are inhibited by G-CSF. While microarray analysis (Dataset in S1 Dataset and Additional Data in S1 Text) suggested that cholinergic receptor nicotinic beta 4 subunit (CHRNB4) may be involved, we were unable to confirm a role for this protein using the ligands acetylcholine or nicotine. Further research on this clinically relevant issue is therefore necessary.

**Table 1:** Patient information for xenografts.

Patient ID	Age(y)/sex	Phenotype	Cytogenetics
1809	5m/F	CD34^+^CD19^+^CD10^+^CD20^+^	46 XY, del(4)(q21q25), -9, add(13)(q14+add(22)(p13)[9/46xy[11]
1345	5/F	CD34^-^CD19^+^CD10^+^CD20^+^	45 XX dup(1)(q42 q25), del (3)(q21), -9, del(9)p22, t(18;20)(q21;q13.1)
2070	65/M	CD34^+^CD19^+^CD10^+^CD20^+^	45 XY t(9;22)(q34;q11.2) del(9)(p21)
2053	4/F	CD34^+^CD10^+^CD19^+^	N/A
0407	45/M	CD34^-^CD19^+^CD10^+^	t(1;19)*
1338	3/M	CD34^+^CD19^+^CD10^+^	46XY
0398	15/M	CD10^-^CD34^+^	46 XY add(3)(q29), t(14;19)(q32;p13)
1999	14/F	CD10^+^CD34^-^	46 XX

* Determined by PCR.

## Supporting information

S1 Dataset Microarray data from ALL xengrafts. Microarray data from two sensitive and four resistant xenografts.
(XLSX)

S1 Text Additional supplementary data. This file contains supplementary Table A, Fig A, Additional Text, Fig B, Table B, Fig C and Fig D.
(DOCX)
