# Supplementary material for: Regulation of the bone marrow microenvironment by G-CSF: Effects of G-CSF on acute lymphoblastic leukaemia
Source: PLoS One. 2017 Nov 16;12(11):e0188042. doi: 10.1371/journal.pone.0188042 (PMC5690634; doi:10.1371/journal.pone.0188042)
Supplement: S1 Text — This file contains supplementary Table A, Fig A, Additional Text, Fig B, Table B, Fig C and Fig D. (DOCX) [file pone.0188042.s002.docx]

**Supporting Information**

**Table A. Additional Patient Information for Xenografts**

| **Patient ID** | ***WCC** | **#% Blasts** | **†Remission/Relapse/Death** |
| --- | --- | --- | --- |
| 1809 | N/A | 94 | 1^st^ Remission >2 years |
| 1345 | 128.1 | 87 | 1^st^ Remission >7 years |
| 2070 | N/A | N/A | N/A |
| 2053 | 50.0 | N/A | 1^st^ Remission >4 years |
| 0407 | 37.1 | 97 | N/A |
| 1338x | 15.4 | 90 | 1^st^ Remission >7 years |
| 0398 | 54.6 | N/A | 1^st^ Relapse 17m, 2^nd^ Relapse 23m, 3^rd^ Relapse 29m, Died 30m. |
| 1999 | N/A | 94 | N/A |

*Values at diagnosis. # Values from diagnostic bone marrow. † Time from diagnosis.

**Figure A. Time course of the engraftment of human ALL cells in the bone marrow NOD/SCID mice.** Mice were electively culled at the indicated time points and the proportion of ALL cells in the bone marrow assessed by flow cytometry.

**
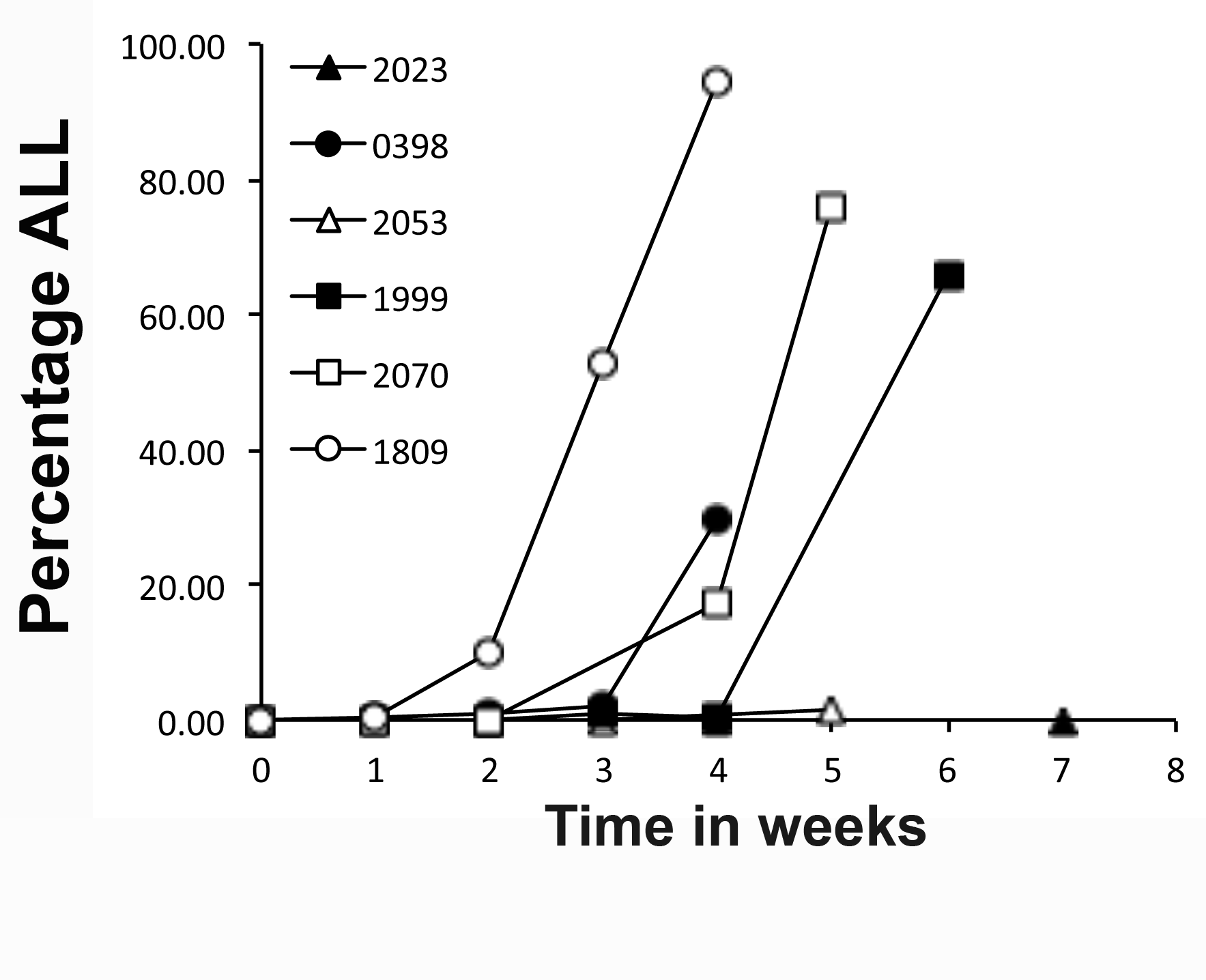
**

**S1 Data File. Microarray Data of Xenografts** is supplied as a separate xlsx file.

**ADDITIONAL DATA**

**Differences in Gene Expression between G-CSF Responsive and Unresponsive Cases.** Global analysis of the microarray data did not identify any genes with increased or decreased expression using a standard 2 fold criteria (Figure 2). However 44 genes were exclusively expressed in the G-CSF responsive cases and significantly higher expression was detected for 65 genes. G-CSF responsive cases lacked expression of 46 genes and a further 142 were expressed at significantly lower level than in the unresponsive cases. There was no significant enrichment for genes of particular function or involvement in specific pathways identified using DAVID gene annotation analysis. We considered surface expressed proteins in more detail, as these are likely to be involved in responses to changes in the microenvironment. The surface expressed proteins either exclusively expressed (restricted expression) or more highly expressed (higher expression) in the responsive xenografts are listed in Table 2. Of these, CHRNB4 has been reported to induce increased cell proliferation and its ligand, acetylcholine, could be produced by bone marrow cells. We found that CHRNB4 was expressed a higher than normal levels (ie greater than 2 standard deviation above the mean) in 15% of pediatric ALL samples collected at the time of diagnosis with those bearing rearrangements in the MLL gene having significantly higher expression (Figure 3). However the biological ligands for CHRNB4, acetylcholine failed to induce proliferative responses in the xenografts that had expanded *in vivo* following G-CSF treatment (Figure 4).

**Figure B. Volcano plot of microarray data from patient xenografts.** No genes were regulated by more than 2 fold. Some of the more highly regulated genes are indicated on the plot.

**
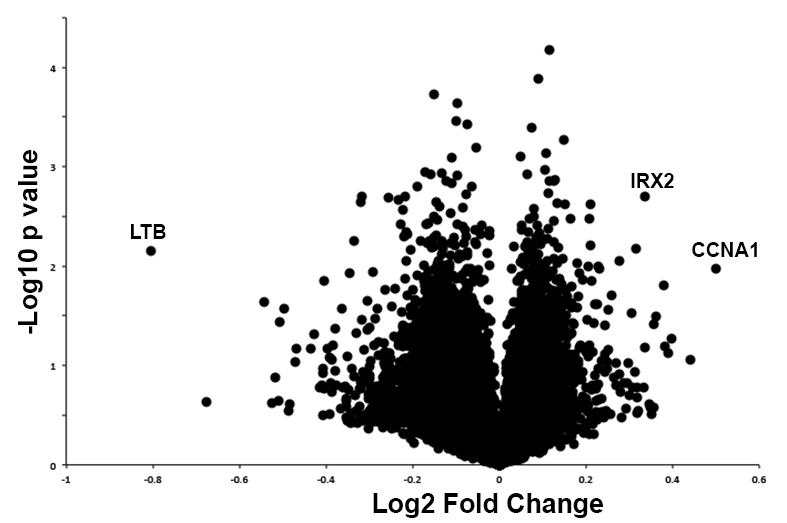
**

**Table B. Gene Expression in G-CSF Responsive Cases**

**Gene Symbol Log 2 Fold Change Description**

**Restricted Expression**

ABCA3 N/A ATP-binding cassette, sub-family A, member 3

CHRNB4 N/A Cholinergic receptor, nicotinic, beta 4 (neuronal)

TMEM117 N/A transmembrane protein 117

PVRL3 N/A Poliovirus receptor-related 3

ASPH N/A aspartate beta-hydroxylase

**Higher Expression**

ABCC4 1.22 ATP-binding cassette, sub-family C, member 4

SLC12A2 1.12 solute carrier family 12 member 2

TLN1 1.06 talin 1

PTPRK 1.38 protein tyrosine phosphatase, receptor type, K

**Figure C. CHRNB4 is expressed in a proportion of pediatric ALL cases at diagnosis.** Analysis of a series of pediatric ALL samples which had been collected at the time of diagnosis from the published dataset GSE28497. The grey shaded region indicates cases expressing above the mean plus 2 standard deviations of the expression found in normal B cell progenitors.


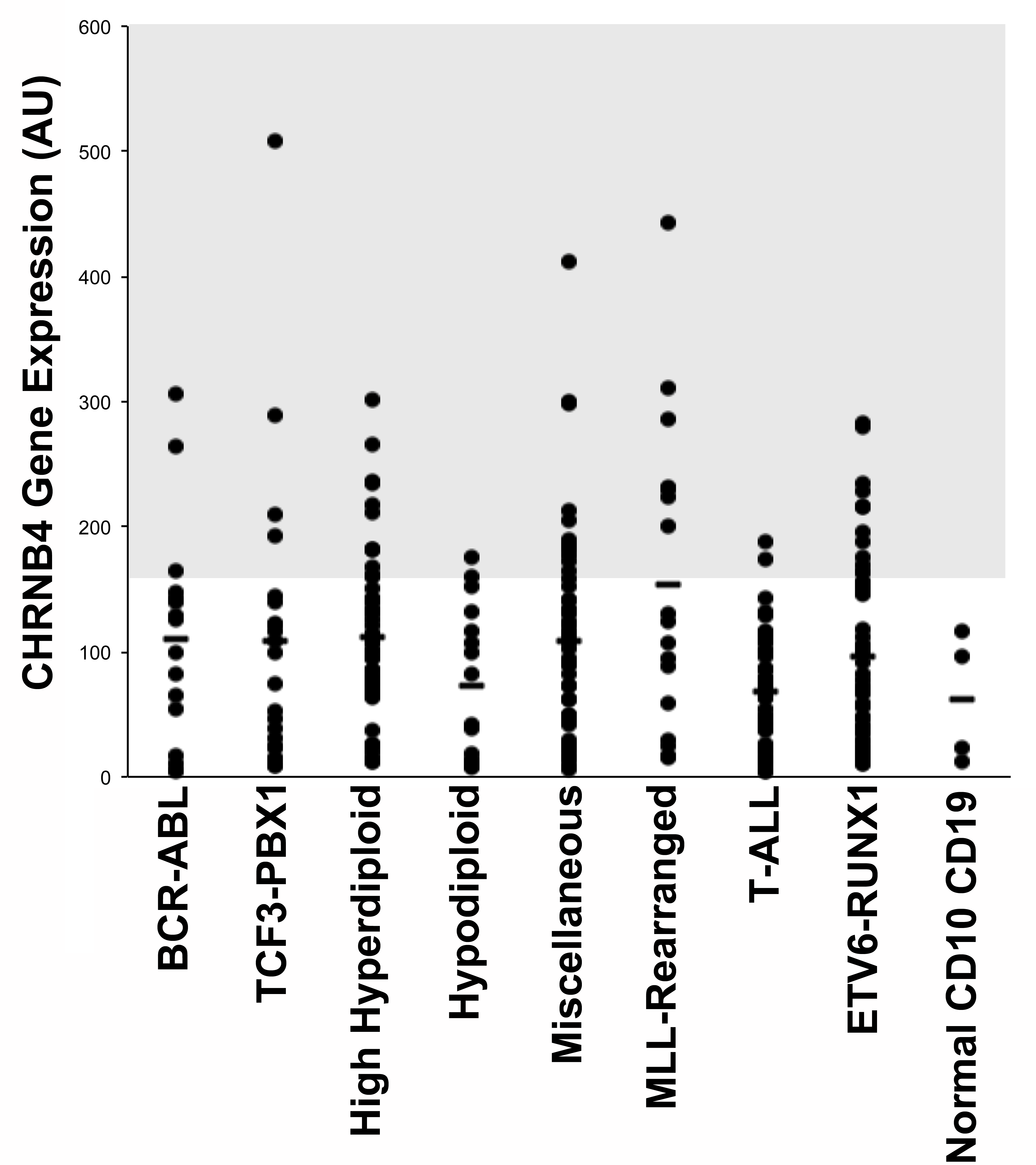


**Figure D. Activation of CHRNB4 does not induce the proliferation of ALL cells.**

ALL cell were cultured with the addition of the biologically relevant CHRNB4 ligand acetylcholine. The data below is from one case and shows the mean and standard deviation of 4 independent experiments performed in quadruplicate. Similar results were obtained using cells from the other case but we only performed 2 experiments. Two alternate ligands nicotine and carbachol produced similar results although the number of experiments was fewer so the data has not been included here.
